# Aggressive motivation mediates the influence of prosocial video game play on young children’s aggressive behavior

**DOI:** 10.3389/fpsyg.2025.1526493

**Published:** 2025-06-04

**Authors:** Yan Li, Tao Deng, Nicola Ngombe, Philipp Kanske

**Affiliations:** ^1^Clinical Psychology and Behavioral Neuroscience, Technische Universität Dresden, Dresden, Germany; ^2^Department of Psychology and Logopedics, University of Helsinki, Helsinki, Finland; ^3^Faculty of Education, Northeast Normal University, Jilin, China

**Keywords:** prosocial video games, aggressive behavior, motivation, pre-schoolers, mediation analysis

## Abstract

**Introduction:**

Aggressive behavior in early childhood can have lasting consequences. This study examined whether prosocial video game play can reduce aggressive behavior in relatively Chinese preschoolers and explored the mediating role of aggressive motivation and the moderating effects of age and gender.

**Methods:**

A total of 132 children aged 4 to 6 years (50% girls; *M* = 5.0, *SD* = 0.82) participated in a between-subjects experiment. Participants were randomly assigned to play either a prosocial or a neutral video game. Aggressive behavior and aggressive motivation were assessed following gameplay.

**Results:**

Children who played the prosocial video game exhibited significantly lower levels of aggressive behavior than those in the neutral game condition. Revenge motivation significantly mediated this effect. The mediating effect was stronger in boys than in girls; age did not moderate the associations.

**Discussion:**

These findings suggest that prosocial video games may be effective in reducing aggressive behavior among preschoolers, partly by lowering revenge-related motivation. Gender differences in the mediation pathway highlight the need for tailored early interventions.

## Introduction

Aggressive behavior is defined as any intentional act aimed at harming another individual (Anderson and Bushman, 2002; Krahé, 2021; McGregor et al., 2015). It is prevalent among children and adolescents, with 46.3% of American students reporting experiences of physical aggression at school (Finkelhor et al., 2009), and 51% of Chinese secondary school students exhibiting high aggression levels (Hu et al., 2023). Notably, even among Chinese preschoolers, 12.4% display aggressive behaviors (Jia et al., 2016), underscoring the need for early interventions.

In 2024, the global gaming population is estimated to reach 3.32 billion, with Asia leading the industry, accounting for approximately 1.48 billion gamers (Gill, 2025), which present a unique opportunity for behavioral interventions. A prosocial video game (PVG) is a game with prosocial content, such as helping, as a theme (Chambers and Ascione, 1987; Gan et al., 2024; Li and Zhang, 2022). Unlike violent or neutral video games, PVGs emphasize altruistic actions where players must engage in prosocial behaviors to progress in the game. PVGs often feature scenarios that encourage empathy, perspective-taking, and conflict resolution, reinforcing socially constructive behaviors (Gan et al., 2024). Research suggests that PVGs can reduce aggression and enhance prosocial tendencies across different age groups, including school-aged children, adolescents, and adults (Greitemeyer, 2011, 2022; Greitemeyer and Osswald, 2009; Saleem et al., 2012). However, little is known about their effects on preschoolers, particularly in China, where aggression is commonly observed in kindergartens. Addressing this gap, the present study investigates the potential of PVGs in reducing aggression in young children.

### Prosocial video games and aggressive behavior

The General Learning Model highlights the role of media content in shaping behavior and suggests that PVG exposure can increase prosocial behavior while reducing antisocial tendencies (Greitemeyer, 2011). According to the General Learning Model, media exposure exerts both immediate (short-term) and long-term effects, depending on the frequency and duration of exposure to similar stimuli (Sarmet and Pilati, 2016). Short-term effects are primarily driven by mechanisms such as priming, arousal, and imitation, whereas long-term effects stem from observational learning, as well as the activation and desensitization of emotional processes (Huesmann, 2007). Supporting this framework, studies have demonstrated that even brief exposure to PVGs can increase prosocial thought accessibility in both children and adults (Greitemeyer and Osswald, 2011; Li and Zhang, 2022).

In relation to aggressive behavior, review studies indicate that while violent video games tend to increase aggression and hinder prosocial outcomes, PVGs have the opposite effect, reducing aggression (Greitemeyer and Mügge, 2014, 2022). Empirical studies further support these findings, showing that PVG exposure reduces harmful behavior in children aged 9–14 years (Saleem et al., 2012), and diminishes hostile expectation bias and antisocial thought accessibility in university students (Greitemeyer and Osswald, 2009). While research has demonstrated the positive impact of PVGs on preschoolers’ prosocial behavior (e.g., Li and Zhang, 2022; Li et al., 2023; Shoshani, 2023), their potential effect on reducing aggression in preschoolers remains uncertain, highlighting the need for further research in this area.

To deepen our understanding of how PVGs influence children’s aggression, it is important to integrate broader socio-emotional constructs into the theoretical framework. In particular, empathy has been widely identified as a key mechanism underlying these effects, as it is strongly associated with both prosocial behavior and the perception of aggression severity (Dou and Zhang, 2025; Gan et al., 2024; Yin and Wang, 2023). PVGs may promote empathy by encouraging perspective-taking and prosocial decision-making, which in turn could reduce children’s aggressive tendencies (Carmona-Cardona et al., 2024; Li and van Berlo, 2025; Yin and Wang, 2023). Moreover, recent studies suggest that prosocial behaviors are associated not only with reduced aggression but also with lower risks of addictive and dysfunctional behaviors (Esparza-Reig et al., 2021; Kasap et al., 2023), positioning these skills as protective factors that support healthy digital engagement. Additionally, resilience and social support have been found to foster the development of prosociality during childhood and adolescence, serving as buffers against emotional and behavioral problems (Esparza-Reig et al., 2022; Luo et al., 2023). Acknowledging their roles provides a more integrative perspective on how social resources interact with game content to influence developmental outcomes. Moreover, as digital environments become increasingly complex, especially for older children and adolescents, recent studies on online prosocial behavior and digital altruism suggest that prosocial tendencies can extend into virtual spaces (Li, 2024; Pastor et al., 2024). These findings offer a valuable direction for future research exploring how PVGs may foster positive social behavior in more interactive digital contexts.

### Aggressive motivation as a possible potential mediator

Research suggests that video game exposure influences aggression through aggressive cognition and aggressive affect (Anderson and Bushman, 2001; Greitemeyer et al., 2012; Greitemeyer and Osswald, 2009). However, the role of aggressive motivation in this process remains less explored. Aggressive motivation—the impulse to harm oneself or others—can be categorized into revenge motivation (harm-driven, emotion-based) and instrumental motivation (goal-oriented, influence-driven) (Lindsay and Anderson, 2000; Lozovska and Gudaitë, 2013). Revenge motivation, also termed hostile aggression, refers to impulsive, emotionally driven behavior, whereas instrumental aggression is calculated, incentive-driven behavior used as a means to an end (e.g., coercion or extortion) (Anderson and Murphy, 2003; Kruglanski et al., 2023).

Previous research highlights the central role of motivation in influencing children’s aggressive behavior (Feshbach, 1964; Harmon-Jones and Schutter, 2022). For instance, revenge motivation has been found to mediate the link between violent video games and aggression (Anderson and Murphy, 2003). According to the General Learning Model, video game features influence a player’s internal state, including motivation, which in turn shapes behavior to align with the game’s content (Sarmet and Pilati, 2016). While motivation is a key factor in the relationship between video game play and aggression, it remains unclear whether aggressive motivation mediates the reduction of aggression through PVG play.

### Gender and age differences

While research supports the potential of PVGs in reducing children’s aggression, their effectiveness may vary based on individual characteristics such as gender and age. Studies consistently indicate that girls exhibit lower overall levels of aggression than boys (e.g., Lansford et al., 2012; Maccoby and Jacklin, 1980; Ticusan, 2014). However, the expression of aggression differs between genders, with boys more likely to engage in physical aggression, such as hitting, while girls tend to display higher levels of relational aggression, such as social exclusion (Björkqvist, 2018; Lansford et al., 2012; Nivette et al., 2020). In addition, gender differences in video game consumption patterns have also been documented: boys generally spend more time on video games and prefer competitive or action-oriented genres, whereas girls may be more drawn to games with prosocial or narrative content (Leonhardt and Overå, 2021; Veltri et al., 2014). Accordingly, research suggests that boys experience greater positive emotional responses during competitive rather than cooperative play (Kivikangas et al., 2014). In contrast, girls appear to be more emotionally responsive to prosocial content in video games (Li et al., 2023). Considering gender-based differences in both aggression expression and game engagement, the associations between PVG play and aggression—as well as the underlying mediating mechanisms—may differ between boys and girls.

On the other hand, age might be a crucial factor shaping children’s response to PVGs. Research suggests that as children grow older, their cognitive and moral development advances, enabling them to better recognize the negative consequences of aggression, which in turn leads to a decline in aggressive behavior (Erfani et al., 2010; Kokko et al., 2006; Underwood et al., 2009). However, early aggression can hinder social development and lead to relationship difficulties and social isolation (Ladd and Troop-Gordon, 2003), highlighting the need for early interventions. Given these developmental differences, it remains unclear whether girls and older children benefit more from PVGs in reducing aggression through decreased aggressive motivation compared to boys and younger children.

### The present study

**FIGURE 1:**
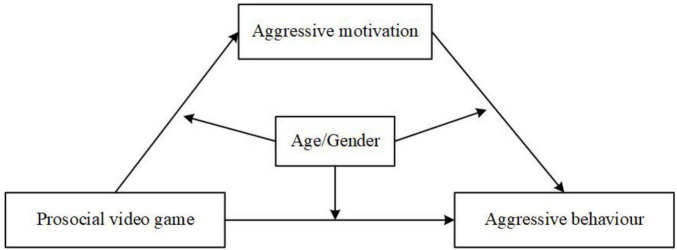
Proposed moderated mediation model.

Existing research on video game effects on children’s aggression reveals several gaps that warrant further investigation. First, while prior studies have demonstrated that PVGs can reduce aggression in school-aged children, college students, and adults (Gentile et al., 2009; Greitemeyer and Osswald, 2009), little is known about their effects on preschool-aged children. Given the developmental differences between preschoolers and older children, it remains unclear whether the aggression-reducing effects of PVGs extend to this younger age group. Second, research has established that aggressive motivation mediates the relationship between violent video games and aggression (Anderson and Murphy, 2003). However, there is limited understanding of how aggressive motivation operates in the context of PVGs. Third, gender differences in aggression are well-documented, with boys exhibiting higher overall levels of aggression, while girls tend to be more responsive to prosocial content in video games (Lansford et al., 2012; Li and Zhang, 2022). However, it remains unclear whether the associations between PVG exposure and aggression differs between boys and girls, particularly in terms of aggressive motivation as a mediating factor. Fourth, as children grow older, their cognitive and moral development advances, allowing them to better recognize the negative consequences of aggression, which in turn leads to a decline in aggressive behavior (Erfani et al., 2010; Kokko et al., 2006; Underwood et al., 2009). However, it is unclear whether older children’s aggression is more susceptible to the influence of PVGs compared to younger children. To address these gaps, this study examines whether PVG exposure reduces aggression in Chinese preschoolers who are relatively more aggressive, with a focus on the mediating role of aggressive motivation (Figure 1). Additionally, it explores whether these mediation effects vary by gender and age. Based on these gaps, this study seeks to explore the following research questions (RQs) and hypotheses (Hs):

RQ1: Does playing a PVG compared to a neutral video game reduce aggressive behavior in preschool children?

• H1: Based on previous findings that PVGs can reduce aggression in older children and adults, we hypothesize that preschoolers who play a PVG will exhibit lower levels of aggressive behavior compared to those who play an NVG.

RQ2: Does aggressive motivation mediate the relationship between PVG play and aggressive behavior?

• H2: Given that aggressive motivation has been shown to mediate the link between violent video games and aggression, we hypothesize that aggressive motivation will mediate the relationship between PVG play and aggressive behavior.

RQ3: Does the mediating effect of aggressive motivation differ between boys and girls?

• H3: Due to limited prior research, we do not propose a specific hypothesis regarding gender differences in the mediating effect but explore this question empirically.

RQ4: Does the mediating effect of aggressive motivation vary by age?

• H4: Considering that older children demonstrate more advanced cognitive and moral development, which may influence how they process prosocial content, we hypothesize that the mediating effect of aggressive motivation between PVG play and aggressive behavior will be stronger for older preschoolers than for younger ones.

## Materials and methods

### Recruitment and participants

The present research utilized teacher nominations to identify aggressive children, a method previously validated for its reliability and precision in gauging children’s aggression (Huesmann et al., 1994). Each teacher was instructed to pinpoint the 10 most aggressive children within their class. To draw clear conclusions about age differences, we specifically selected children close to a certain age, namely within an age range of ± 2 months. We recruited 136 relatively aggressive children from two kindergartens in Chongqing, China. Four of these selected children declined to participate in the experiment. Therefore, the final sample consisted of 132 pre-schoolers aged between 4 and 6 years (50% girls, 50% boys; M_*age*_ = 5.0, SD = 0.82), who were randomly assigned to play prosocial or neutral video games individually for 15 min. Sixty-six pre-schoolers (girls = 33; *N*_4–*years*–*old*_ = 22, *N*_5–*years*–*old*_ = 22, *N*_6–*years*–*old*_ = 22) were randomly assigned to play the PVG, while the other 66 pre-schoolers (girls = 33; *N*_4–*years*–*old*_ = 22, *N*_5–*years*–*old*_ = 22, *N_6–year*s*–olds_* = 22) played the neutral video game. None of the participants had an intellectual disability.

### Measurements

#### Video games

The game “Lemmings” was selected as the PVG for this study, consistent with its use in prior research exploring the impacts of PVGs (Greitemeyer and Osswald, 2009). In Lemmings, players guide groups of human-like lemmings through different worlds, trying to save them by leading them to the exit. Tetris was used as the neutral video game and it required players to move, rotate and arrange different descending blocks into a complete line without gaps in order to receive points (Atmadji et al., 2023). Participants were randomly assigned to play the prosocial or neutral video game for 15 min.

#### Competitive reaction-time task

The competitive reaction-time task (CRT) is a valid laboratory measure of aggression (Anderson and Murphy, 2003; Warburton and Bushman, 2019). Participants compete against a virtual opponent to see who can respond fastest to the tone. At the end of each trial, the “loser” is punished with a loud noise set by their opponent. The pattern of wins/losses and the level of noise imposed on participants in the “lose” trials are predetermined. In this study, we used a two-session version of the task. Session 1 consisted of 25 trials in which the supposed “opponent” set the noise level for the participant in the “lose” trials. Trial 1 was a “win.” The remaining 24 trials were segmented into three blocks, each containing eight trials. In every block, participants experienced an equal distribution of outcomes, with four wins and four losses. Therefore, in Session 1, each participant encountered a consistent pattern of outcomes, totaling 13 wins and 12 losses. After completing Session 1, the experimenter reminded the participants that in Session 2 they would set the noise intensities for their opponent. Session 2 was identical except that the roles were reversed. Prior to Session 1, participants were given example noise levels of “1” (60 dB) and “5” (100 dB). The indicator of aggressive behavior is the level of noise intensity (noise levels ranged from 1 to 5, with loudness ranging from 60 to 100 dB, respectively) that participants set for their opponents. A no-noise option (0 dB, no aggressive behavior) was available.

#### Aggressive motivation

Following the CRT, participants completed the motivation questionnaire (Anderson and Murphy, 2003). We used six items to ask participants to indicate the extent to which motivation played a role in their decision to increase the noise level (Anderson and Carnagey, 2009). Two items measured instrumental motivation (e.g., “I wanted to control my opponent’s level of responses”) and four items measured revenge motivation (e.g., “I wanted to hurt my opponent”). Responses were captured on a five-point Likert scale ranging from 1 (strongly disagree) to 5 (strongly agree). Cronbach’s alpha for the total aggressive motivation score was 0.72 and for instrumental motivation and revenge motivation 0.82 and 0.90, respectively.

### Procedure

Before the experiment, participants were informed that they could decline participation without any negative consequences during experiment process. Written informed consent was obtained from their parents or legal guardians, and the children also provided verbal assent. Participants were randomly assigned to play the PVG or neutral video game individually for 15 min. Afterward, they completed the CRT with the understanding that they would have no contact with their virtual opponents. Post-CRT, participants were debriefed about their aggressive motivation. All procedures were then explained, questions were answered, and participants were thanked. For children with limited literacy skills, verbal instructions were provided for the motivation questionnaire, supplemented by examples if necessary. Participants understood how to adjust noise intensity upon winning and the motivation questionnaire’s content. The study was conducted according to the guidelines of the Declaration of Helsinki and approved by the Ethics Committee, Faculty of Education, Northeast Normal University (20200601.02 and date of 1 June 2020).

### Data analysis

Firstly, in order to know whether PVG play will influence children’s aggressive behavior (RQ1), we performed an independent samples *t*-test to compare the levels of aggressive behavior between the PVG and the control group. Next, we employed mediation models to investigate the potential mediating influence of aggressive motivation (RQ2) (Chen et al., 2005). Additionally, we explored the moderating effects of age and gender using a moderated mediation analysis approach (RQ3 and RQ4), following the methodology outlined in prior studies (Preacher et al., 2007). For both the mediation and moderated mediation models, we utilized bootstrapping (Preacher and Hayes, 2004) as a robust method to obtain reliable standard errors (SEs) for parameter estimation. Power analyses in G*Power and WebPower with an α-level of.05 indicated adequate statistical power for *t*-test and mediation analysis (1–β) > 0.80.

## Results

### Differences between neutral and prosocial video game groups

**FIGURE 2:**
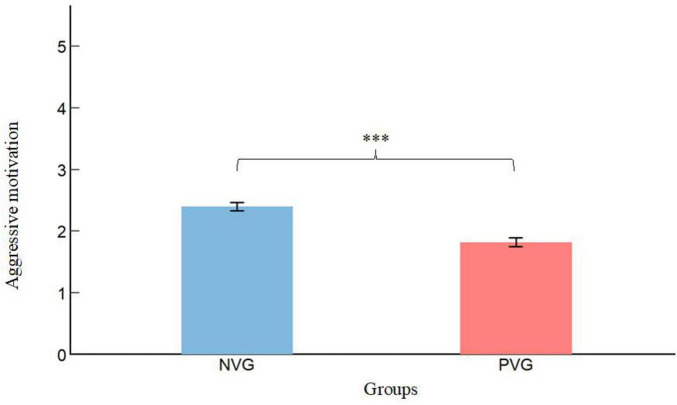
Group differences in aggressive motivation. NVG, neutral video game; PVG, prosocial video game. Error bars represent standard errors. ***p < 0.001.

**FIGURE 3:**
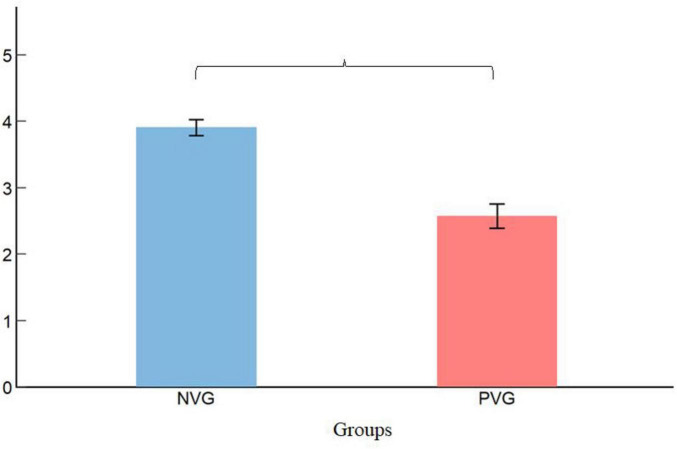
Group differences in aggressive behavior. NVG, neutral video game; PVG, prosocial video game. Error bars represent standard errors. ***p < 0.001.

Table 1 presents the means and standard deviations of aggressive behavior and aggressive motivation across 12 subgroups. As shown in Figure 2, the *t*-tests results revealed significant differences between children who played the PVG (M = 1.83, SD = 0.59) and those who played the neutral video game (M = 2.40, SD = 0.54) in terms of aggressive motivation [t (130) = 5.79, *p* < 0.001, d = 1.01]. Accordingly, as presented in Figure 3, the PVG group (M = 2.58, SD = 1.56) exhibited significantly lower levels of aggressive behavior compared with the neutral video game group (M = 3.91, SD = 1.01), t (130) = 7.89, *p* < 0.001, d = 1.01.

**TABLE 1:** Means and standard deviations of aggressive motivation and aggressive behaviour.

Game	Age	Aggressive motivation				Aggressive behavior			
		Boys (50%)		Girls (50%)		Boys (50%)		Girls (50%)	
		M	SD	M	SD	M	SD	M	SD
Prosocial	4 years-old	2.24	0.53	2.12	0.40	3.91	0.83	1.55	1.04
	5 years-old	2.06	0.64	1.71	0.070	2.82	1.33	2.09	1.45
	6 years-old	1.52	0.32	1.32	0.27	1.00	0.78	1.18	1.08
Neutral	4 years-old	2.42	0.58	1.95	0.45	4.18	0.75	3.73	0.91
	5 years-old	2.76	0.43	2.50	0.34	4.18	0.60	3.64	1.12
	6 years-old	2.23	0.43	2.53	0.67	3.36	1.36	3.73	0.79
Total	–	2.20	0.61	2.02	0.64	3.24	1.47	2.65	1.50

Number of participants = 132.

### Aggressive motivation as a potential Mediator of aggression

**FIGURE 4:**
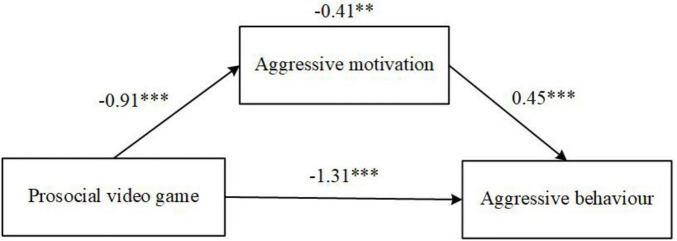
Mediation model of prosocial video games on aggressive behavior through aggressive motivation. **p < 0.01; ***p < 0.001.

The mediation model revealed that aggressive motivation significantly mediated the relationship between playing the PVG and aggressive behavior [β = –0.41, SE = 0.13, 95% CI (–0.67, –0.16)], indicating that the effect of PVG exposure on aggression operates, at least in part, through changes in aggressive motivation. As illustrated in Figure 4, PVG play was a significant predictor of aggressive motivation [β = 0.91, SE = 0.16, 95% CI (–1.21, –0.59)]. suggesting that exposure to prosocial gaming reduced children’s aggressive motivation. In turn, aggressive motivation was positively associated with aggressive behavior [β = 0.45, SE = 0.12, 95% CI (0.22, 0.68)], confirming that children with higher aggressive motivation exhibited more aggressive behaviors. Furthermore, a direct effect of PVG play on aggressive behavior remained significant [β = –1.31, SE = 0.23, 95% CI (–1.76, –0.84)], indicating that beyond its indirect effects through aggressive motivation, PVG play had a direct influence in reducing aggression in preschool children.

**FIGURE 5:**
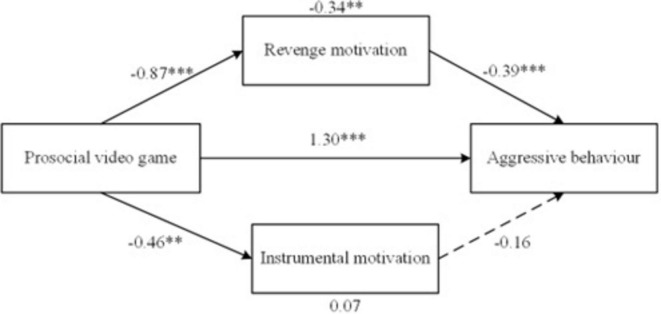
Mediation model effect of prosocial video games on aggressive behavior through revenge motivation and instrumental motivation. Solid lines represent significant paths, dashed lines represent non-significant paths. **p < 0.01, ***p < 0.001.

Furthermore, we tested whether revenge motivation and instrumental motivation mediated the effects of PVG play on aggressive behavior. As shown in Figure 5, revenge motivation mediated the association between PVG play and aggressive behavior [β = –0.34, SE = 0.12, 95% CI (–0.59, –0.13)], suggesting that reductions in revenge-driven aggression contribute to the overall decrease in aggression following PVG exposure. Playing the PVG significantly predicted lower levels of revenge motivation [β = –0.87, SE = 0.16, 95% CI (–1.18, –0.56)], and then reduced revenge motivation, in turn, significantly predicted lower levels of aggression [β = –0.39, SE = 0.12, 95% CI (0.16, 0.62)]. However, instrumental motivation did not significantly predict aggressive behavior [β = 0.07, SE = 0.05, 95% CI (–0.19, 0.03)], indicating that children’s use of aggression for goal-oriented purposes was not a key explanatory mechanism in this context. Playing the PVG significantly predicted instrumental motivation [β = –0.46, SE = 0.17, 95% CI (–0.80, –0.13)], but instrumental motivation did not predict aggressive behavior [β = –0.16, SE = 0.11, 95% CI (–0.06, 0.37)]. Overall, the reducing effect of PVG play on aggressive behavior was partly mediated by aggressive motivation, especially revenge motivation.

### Moderating effect of gender and age

**FIGURE 6:**
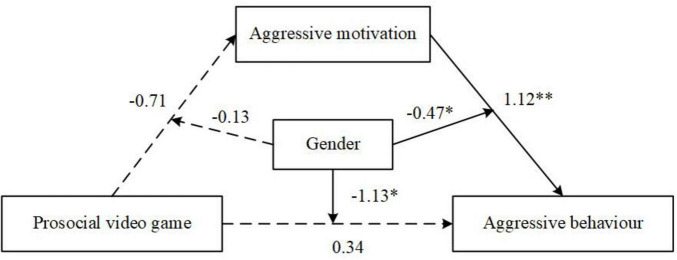
Moderated mediation model with gender as a moderator. Solid lines represent significant paths, dashed lines represent non-significant paths. *p < 0.05, **p < 0.01.

The moderated mediation analysis revealed that gender significantly moderated the mediating effect of aggressive motivation, indicating that the indirect pathway from PVG play to aggressive behavior via aggressive motivation differed between boys and girls, as shown in Figure 6. Specifically, the mediating effect of aggressive motivation was significant for boys [β = –0.54, SE = 0.19, 95% CI (–0.94, –0.20)], but not for girls [β = –0.16, SE = 0.16, 95% CI (–0.47, 0.18)]. Gender moderated the effect of PVG play on aggressive behavior [β = –1.13, SE = 0.45, 95% CI (–2.02, –0.25)] and the effect of aggressive motivation on aggressive behavior [β = –0.47, SE = 0.23, 95% CI (–0.92, –0.02)]. Similarly, the mediating effect of revenge motivation was also only significant for boys [β = –0.43, SE = 0.18, 95% CI (–0.84, –0.12)] and not for girls [β = –0.16, SE = 0.15, 95% CI (–0.49, 0.14)]. These findings suggest that the link between aggressive motivation and aggression is stronger for boys,

In contrast with H4, the mediation effect was not significant at ages 4 [β = –0.04, SE = 0.08, 95% CI (–0.19, 0.13)], 5 [β = –0.28, SE = 0.13, 95% CI (–0.52, 0.01)] or 6 years [β = –0.56, SE = 0.42, 95% CI (–1.36, 0.29)], indicating that while PVGs influence aggression through motivational changes, these effects do not vary meaningfully with age in the preschool years.

In addition, age did not significantly moderate the impact of PVG play on aggressive behavior [β = –0.36, SE = 0.29, 95% CI (–0.93, 0.21)] or that of aggressive motivation on aggressive behavior [β = –0.03, SE = 0.15, 95% CI (–0.27, 0.33)]. Likewise, age failed to moderate the mediation effect of revenge motivation, which was not significant for children aged 4 [β = –0.05, SE = 0.10, 95% CI (–0.29, 0.13)], 5 [β = –0.26, SE = 0.14, 95% CI (–0.53, 0.003)] and 6 years [β = –0.38, SE = 0.44, 95% CI (–1.23, 0.45)].

Overall, gender moderated the mediating effect of aggressive motivation between PVG play and aggressive behavior, with a stronger effect observed for boys. This trend was also observed for the mediating effect of revenge motivation. However, the mediating effect of age was not significant.

## Discussion

The current study found that aggressive preschoolers in the sample who played a PVG exhibited significantly lower levels of aggression compared to those who played a neutral game. The effect was mediated by aggressive motivation, particularly revenge motivation, and was more pronounced in boys than girls, with no significant differences across preschool age groups.

In line with H1, children exposed to PVG play demonstrated lower aggression, supporting the General Learning Model, which posits that game content shapes behavior (Gentile et al., 2014; Saleem et al., 2012). Prior research suggests that video games can have both positive and negative social effects depending on their content (Greitemeyer and Mügge, 2014). In this study, prosocial elements (e.g., helping others) likely enhanced prosocial cognition while inhibiting antisocial thoughts, reducing aggressive behavior. These findings highlight PVGs as a potential intervention tool for educators and parents seeking to reduce aggression in preschoolers. Future studies could examine how developmental and environmental factors shape the effects of PVGs on aggression, offering deeper insights into their potential as an early intervention strategy for reducing aggressive behavior in preschoolers.

In line with the H2, playing the PVG was found to decrease aggression by mediating aggressive motivation, particularly revenge motivation. This finding aligns with the broader literature on the potential of PVGs to diminish aggressive cognition, emotions and hostile attributions (Gentile et al., 2009; Greitemeyer and Osswald, 2009). In line with the General Learning Model, game content shapes players’ internal states, which in turn influence behavioral outcomes (Greitemeyer et al., 2012). The presence of prosocial cues in PVGs may counteract aggressive motivation, thereby lowering children’s aggressive behaviors. Notably, revenge motivation, recognized as a key driver of harmful intent (León-Moreno et al., 2019), emerged as a significant mediator in the relationship between PVG exposure and aggression. This finding suggests that engaging with PVGs may reduce children’s inclination toward retaliatory thinking, potentially decreasing their aggressive behavior. From an applied perspective, interventions leveraging PVGs as a tool to mitigate aggression should prioritize targeting children’s aggressive motivation, with a particular focus on diminishing revenge-driven responses. The current study extends prior work by providing empirical support for revenge motivation as a key mechanism underlying aggression reduction. This highlights the potential for PVGs to influence specific motivational processes related to aggression, rather than merely reducing overall aggression levels. Future research could investigate whether similar mechanisms, e.g., emotional regulation or empathy, operate across different populations, particularly in longitudinal designs that examine the stability of these effects over time.

Regarding gender difference, the mediating role of aggressive motivation between PVG play and reduced aggression was significant only for aggressive boys but not for aggressive girls. This finding initiates a complex discussion related to the nature of aggression, gender and the influence of PVGs. PVGs often present scenarios that rewards cooperation, kindness and empathy, in direct contrast to aggression (Gentile et al., 2009). When boys, who statistically tend to exhibit more physically aggressive behaviors (Nivette et al., 2020), play these games, they are exposed to these prosocial values in an engaging and interactive manner. Therefore, PVGs may have a strong influence on boys who are often observed to have greater aggressive tendencies, potentially leading to a more significant reduction in aggressive motivation and behavior. In addition, it is also possible that girls, who have been suggested to exhibit more relational or indirect forms of aggression (Hess and Hagen, 2006), might not be influenced by these video games in the same way as boys. More specifically, it may be that the PVGs do not sufficiently address the specific forms of aggression commonly exhibited by girls, which offers a potential explanation for the non-significant findings. Future studies might benefit from examining the impact of PVGs on different forms of aggression and tailoring interventions to specifically target these forms of aggression in both genders.

Interestingly, the findings revealed a consistent mediating effect of aggressive motivation between PVG play and reduced aggression across children aged 4, 5, and 6 years, contrary to H4. This suggests that PVG exposure influences aggressive behavior uniformly, despite developmental differences in early childhood. Several factors may explain this pattern. First, while cognitive and emotional development advances rapidly during this period, foundational social skills such as cooperation, empathy, and understanding of consequences are already emerging by age four (Kochanska et al., 2000). PVGs, which emphasize prosocial actions, may reinforce these skills consistently across these ages. Second, the design of PVGs, with narratives centered on cooperation and empathy, may provide a universally accessible framework that influences children similarly throughout early childhood. Finally, the study’s modest sample size may have limited the detection of nuanced age-related differences in the moderating effect, warranting further investigation with larger samples.

While our findings support the role of PVGs in reducing aggression, their applicability across different cultural and educational contexts requires further examination. Cultural norms and socialization processes influence children’s responses to media content, including video games (Knobloch et al., 2006). For instance, collectivist cultures may emphasize group harmony and conflict avoidance, potentially amplifying the prosocial effects of PVGs, whereas individualistic cultures might yield different patterns of engagement and aggression reduction (Bergmüller, 2013; Lim, 2009). Future studies could explore whether the mechanisms identified in our study, particularly the mediating role of revenge motivation, operate similarly across diverse cultural settings. Additionally, educational environments play a crucial role in shaping children’s behavioral outcomes. School curricula, classroom dynamics, and teacher-student interactions can either reinforce or diminish the impact of PVGs on aggression reduction (Pianta et al., 2002; Rutter, 1980). Integrating PVGs into structured educational programs may enhance their effectiveness, particularly in settings that emphasize social-emotional learning and conflict resolution. However, the extent to which PVGs can be effectively incorporated into different educational frameworks remains an open question. Further research should investigate how variations in teaching methods, digital literacy, and school policies affect the generalizability of these findings across diverse educational contexts.

## Limitations and implications

The present study provided some important and promising insights into the mechanisms underlying the beneficial effects of PVG play on reducing aggressive behavior in 4–6 years-old Chinese children who are relatively more aggressive. By expanding the theoretical understanding of how PVGs influence aggressive motivation, this study offers meaningful implications for designing intervention strategies that leverage game-based approaches to foster prosocial behavior and reduce retaliatory aggression in children. Nevertheless, there are several limitations that should be addressed in future studies. First, although teacher nomination is validated to select aggressive participants (Huesmann et al., 1994), it may not be the most accurate method of identifying the most aggressive children. Future research could use multi-method approaches, such as combining self-reports, teacher ratings and behavioral observations, to improve the accuracy of aggression measures. Second, the selection of video game materials could be further refined. *LeMMings*, used as the PVG stimulus in this study, was originally designed for slightly older children and may pose cognitive challenges for preschoolers. While we mitigated this by selecting beginner levels, providing clear instructions, and allowing time for familiarization, future research could explore more age-appropriate games, such as *Dora Saves the Dog* (Li and Zhang, 2022). Similarly, *Tetris*, used as the neutral video game, may have imposed cognitive demands that could lead to frustration, potentially influencing aggression scores (Kruglanski et al., 2023). Although we addressed this by using beginner levels and allowing familiarization time, future studies should consider alternative neutral games with lower cognitive load, such as *Bear Baba*, to better isolate the effects of PVG exposure (Li and Zhang, 2022). Third, this study used the CRT to measure aggression in preschoolers. Considering its competitive nature and cognitive demands may not fully align with their developmental stage. Future research could consider complementing or replacing it with more ecologically valid measures of aggression, such as the hot sauce paradigm (Adachi and Willoughby, 2011). In addition, behavioral observations or informant reports from parents or teachers may better capture real-world expressions of aggression in this age group. Finally, although the experimental design enhances the internal validity of the findings, it does not capture the durability of the observed effects. Future longitudinal research is needed to determine whether the prosocial benefits of PVG play are maintained over time and whether they extend beyond the experimental setting to influence real-world social behavior.

## Conclusion

In conclusion, this study provides preliminary evidence that short-term exposure to prosocial video games may reduce aggressive behavior in preschool-aged children, potentially through a decrease in aggressive motivation—particularly revenge motivation. Moreover, the associations between PVG play, aggression, and the underlying mediating mechanisms appeared to be more pronounced in boys than in girls, while no significant age-related differences were observed among preschoolers. Future research should explore whether the observed effects are maintained over time and whether similar mediating mechanisms operate across different developmental stages and cultural contexts.

## Data Availability

The raw data supporting the conclusions of this article will be made available by the authors, without undue reservation.
